# Mitochondrial uncouplers inhibit hepatic stellate cell activation

**DOI:** 10.1186/1471-230X-12-68

**Published:** 2012-06-11

**Authors:** Eduardo L Guimarães, Jan Best, Laurent Dollé, Mustapha Najimi, Etienne Sokal, Leo A van Grunsven

**Affiliations:** 1 Department of Cell Biology, Liver Cell Biology Lab, Vrije Universiteit Brussel, Laarbeeklaan 103, Brussels, 1090, Belgium; 2 Laboratory of Pediatric Hepatology and Cell Therapy, Université Catholique de Louvain (UCL), Brussel, Belgium

**Keywords:** Hepatic stellate cell, Mitochondria, Uncoupler, Fibrosis

## Abstract

**Background:**

Mitochondrial dysfunction participates in the progression of several pathologies. Although there is increasing evidence for a mitochondrial role in liver disease, little is known about its contribution to hepatic stellate cell (HSC) activation. In this study we investigated the role of mitochondrial activity through mild uncoupling during *in vitro* activation of HSCs.

**Methods:**

Cultured primary human and mouse HSCs were treated with the chemical uncouplers FCCP and Valinomycin. ATP levels were measured by luciferase assay and production of reactive oxygen species was determined using the fluorescent probe DCFH-DA. Possible cytotoxicity by uncoupler treatment was evaluated by caspase 3/7 activity and cytoplasmic protease leakage. Activation of HSCs and their response to the pro-fibrogenic cytokine TGF-β was evaluated by gene expression of activation markers and signal mediators using RT-qPCR. Proliferation was measured by incorporation of EdU and protein expression of α-smooth muscle actin was analyzed by immunocytochemistry and western blot.

**Results:**

FCCP and Valinomycin treatment mildly decreased ATP and reactive oxygen species levels. Both uncouplers increased the expression of mitochondrial genes such as Tfam and COXIV while inducing morphological features of quiescent mouse HSCs and abrogating TGF-β signal transduction. Mild uncoupling reduced HSC proliferation and expression of pro-fibrogenic markers of mouse and human HSCs.

**Conclusions:**

Mild mitochondrial uncoupling inhibits culture-induced HSC activation and their response to pro-fibrogenic cytokines like TGF-β. These results therefore suggest mitochondrial uncoupling of HSCs as a strategy to reduce progression of liver fibrosis.

## Background

Hepatic stellate cells (HSCs) are liver pericytes, located between hepatocytes and sinusoidal endothelial cells, participating in different aspects of liver physiology, with a fundamental function in vitamin A metabolism [1]. HSCs are extensively studied due to its role as the major extracellular matrix producing cell upon hepatic injury, playing a key role in the progression of chronic liver diseases. In the healthy liver it presents a so-called quiescent phenotype, containing lipid droplets rich in retinol and with a balanced extracellular matrix production. Upon chronic liver injury HSCs activate, a process where the cell loses its quiescent phenotype and acquires an activated myofibroblastic one. This phenotype presents a high proliferation rate, produces large amounts of extracellular matrix and inflammatory cytokines, stimulating a positive feedback of inflammatory cell recruitment and collagen deposition [2].

Mitochondria are well established contributors to alcohol induced liver disease. For instance, chronic alcohol intake reduces ATP levels in hepatocytes through decreased translation of mitochondrial proteins or damaging mitochondrial DNA, further increasing cell damage [3,4]. Recently, mitochondria activity has also been recognized as a possible factor in the development of non-alcoholic fatty liver disease (NAFLD) [5]. One of the first links between NAFLD and mitochondria disturbance came from studies showing that obesity induces the expression of uncoupling proteins in hepatocytes, which decrease the mitochondrial proton gradient and hepatic ATP levels [6]. Reduced ATP levels can sensitize and induce hepatocyte apoptosis and aggravate liver injury [5].

Even though mitochondrial uncoupling and ATP depletion is associated with different pathologies, several studies indicate that mild uncoupling, a modest decrease in ATP levels, can elicit a number of physiological beneficial effects when applied to diseases related to obesity, such as diabetes and NAFLD. For example, chemical uncouplers, molecules that stimulate the leakage of protons through the mitochondrial inner membrane, are known to induce mitochondrial biogenesis, a process that ameliorates diabetes type 2 [7]. Mild mitochondrial uncoupling also inhibits lipid accumulation in adipocytes, indicating such mechanism as an appealing anti-obesity strategy [8]. Interestingly, mild chemical uncoupling can mimic the effects of calorie restriction, increasing life span and reducing reactive oxygen species (ROS) levels in mice [9]. Although the consequences of mitochondrial activity have been extensively studied in hepatocytes, little is known about its role in HSC physiology. We studied the effect of mild mitochondrial uncoupling in HSCs using the two different chemical uncouplers Carbonyl cyanide-p-trifluoromethoxyphenylhydrazone (FCCP) and Valinomycin. We show that mild chemical uncoupling is able to reduce several aspects of HSC activation indicating that HSC mitochondrial metabolism may be a new target in the prevention of liver fibrosis.

## Methods

### Isolation of mouse and human hepatic stellate cells

Animals were used in accordance with institutional ethical guidelines. The mouse HSC isolation method, from approximately 20 week old male mice (30 grams), used in this study was previously described [10]. Human HSCs were isolated as follows: human liver non-parenchymal cells were obtained from the left liver segment originating from a healthy cadaveric donor. These cells were obtained after two step collagenase perfusion of the liver segment, filtration and two low speed centrifugations of the cell suspension [11]. Next, human HSCs were isolated by nycodenz (Myegaard, Oslo, Norway) gradient centrifugation. For protein analyzes, 250 × 10^3^ human HSCs were plated in 10 cm^2^ dishes, while 40 × 10^3^ and 20 × 10^3^ were plated in 6 well and 24 well plates for RNA and proliferation analyzes, respectively. The protocol and experiments were approved by the ethical committees of the St-Luc Hospital and faculty of Medicine of Université Catholique de Louvain.

After isolation, human and mouse cells were cultured in Dulbecco’s modified Eagle’s medium with 10% fetal bovine serum at 37°C, in a humidified atmosphere with 5% CO_2_. Treatment with chemical uncouplers or DMSO as a vehicle was performed from day one of culture and after every two days, until cells were collected for the different assays. Bright field images were taken with an Axioskop light microscope (Carl Zeiss, Zaventem, Belgium).

### RNA expression

After treatment with uncouplers and transforming growth factor-β (TGF-β) (R&D Systems), total RNA was extracted using the RNeasy Mini-kit (Qiagen, Hilden, Germany). RNA was reverse-transcribed using the RevertAid™ Premium Reverse Transcriptase (Fermentas, St. Leon-Rot, Germany), performed at 25°C for 10 minutes and at 37°C for 40 minutes. For human HSCs, Maxima® SYBR green qPCR Master Mix (Fermentas) was used. For the semi-quantitative PCR of mouse HSCs we used an Applied Biosystems 7500 Real-Time PCR System (Applied Biosystems, Foster City, CA, USA). Primers (view Table 1 for details) were produced by Invitrogen (Paisley, UK) and probes by Roche (universal probe library, Mannheim, Germany). A 2x Maxima Probe qPCR Master Mix was used (Fermentas, St. Leon-Rot, Germany) to analyze gene expression as described previously [12].

**Table 1:** Primer sequences, probes and accession numbers of transcripts, used for RT PCR quantification

** Gene**	**Primers sequence (Left primer/Right primer)**	**Probe**	**GenBank accession number**	**Product lenght**
18S	5′-aaatcagttatggttcctttggtc-3′/	55	AY_248756	67
	5′gctctagaattaccacagttatccaa-3′			
PGC1α	5′- cagtcgcaacatgctcaag-3′/	6	NM_008904	73
	5′- tggggtcatttggtgactct-3′			
Tfam	5′- caaaggatgattcggctcag-3′/	97	NM_009360	92
	5′- aagctgaatatatgcctgcttttc-3′			
CoxIV	5′- tcactgcgctcgttctgat-3′/	7	NM_37829.1	67
	5′- cgatcgaaagtatgagggatg-3′			
Ndufs7	5′- gtggtgaccaagctggatg-3′/	104	NM_029272	67
	5′- cgaaggtcataggccacag-3′			
HO-1	5′- gtcaagcacagggtgacaga-3′/	4	NM_010442	77
	5′- atcacctgcagctcctcaaa-3′			
Smad6	5′- gttgcaacccctaccacttc -3′/	70	NM_008542	76
	5′- ggaggagacagccgagaata -3′			
Smad7	5′- acccccatcaccttagtcg -3′/	63	NM_001042660	75
	5′- gaaaatccattgggtatctgga -3′			
α-SMA	5′-ccagcaccatgaagatcaag-3′/	58	NM_007392	70
	5′-tggaaggtagacagcgaagc-3′			
Pdgfrβ	5′-tgcagagacctcaaaaggtg-3′/	63	NM_008809.1	112
	5′- cctgatcttcctcccagaaa-3′			
Procol1a1	5′-acctaagggtaccgctgga-3′/	19	NM_007742	97
	5′-tccagcttctccatctttgc-3′			
α-SMA*	5′-ctgttccagccatccttcat-3′/		NM_001141945	70
	5′-tcatgatgctgttgtaggtgg-3′			
PROCOL1A1*	5′-gacacagaggtttcagtgg-3′/		NM_000088.3	264
	5′-cacccttagcaccaacag-3′			
18S*	5′-aagacggaccagagcgaaag-3′/		K03432	98
	5′-tcggaactacgacggtatct-3′			
PDGFR-β*	5′-cccttatcatcctcatcatgc-3′/		NM_002609.3	60
	5′-ccttccatcggatctcgtaa-3′			

*18S* 18S ribosomal RNA, *PGC1 α* Peroxisome proliferative activated receptor gamma, coactivator 1α, *Tfam* Transcription factor A, mitochondrial, *CoxIV* Cytochrome c oxidase subunit IV, *Ndufs7* NADH dehydrogenase (ubiquinone) Fe-S protein 7, *HO-1* Heme oxygenase-1, *Smad6* MAD homolog 6, *Smad7* MAD homolog 7, *α-SMA* α-smooth muscle actin, *Pdgfrβ* Platelet-derived growth factor receptor β, *Procol1a1* Procollagen 1a1. * primers for human samples.

### Western blotting

Cells were exposed to lysis buffer (170 mM NaCl, 10 mM EDTA, 50 mM Tris pH 7.4, 50 mM NaF, 0.2 mM dithiothreitol and 0.5% NP-40) plus protease and phosphatase inhibitors. Protein concentration was measured using a bicinchoninic acid (BCA) determination kit (Pierce Chemical Co., Rockford, IL, USA). Ten microgram of protein was separated on 12% Tris–glycine SDS-Polyacrylamide gels and transferred onto polyvinyldifluoride (PVDF) membranes (Amersham Biosciences, Little Chalfont, UK) using a semidry blotting apparatus (Apollo^TM^, Continental Lab Products, San Diego, CA, USA). Following, membranes were blocked in 5% milk in TBS-Tween. After overnight incubation with primary antibodies at 4°C (anti-α-smooth muscle actin, 1/1000; anti-β-actin, 1/10000, both from Sigma, St Louis, MO, USA) and 1 hour incubation with horseradish peroxidase conjugated secondary antibodies (1/20000) (Dako, Glostrup, Denmark), proteins were visualized with the ECL chemiluminescence detection system (Pierce Chemical Co.). Densitometry analysis was performed using Image J.

### Immunocytochemistry

Freshly isolated mouse and human HSCs were cultivated on glass cover slips in a 24 well plate and fixed with 4% paraformaldehyde for 10 minutes. Cells were permeabilized by 0.1% Triton-X 100 (in PBS with 1% bovine serum albumin) for 30 minutes followed by washing. Mouse HSCs were incubated with monoclonal antibody against α-Smooth Muscle actin (α-SMA) coupled with Cy3 while human HSCs were incubated with unlabeled anti-α-SMA (both from Sigma-Aldrich, St. Louis, MO, USA), at 4°C, overnight. After washing with PBS, human HSCs were incubated with secondary antibody conjugated with Alexa 488 (1:300) (Molecular Probes, Eugene, USA) for 1 hour at room temperature. Following washing and mounting with ProLong® Gold antifade reagent with DAPI (Invitrogen), cells were analyzed by fluorescent microscopy (Carl Zeiss, Zaventem, Belgium).

### Measurement of ROS

Intracellular ROS levels were determined by measuring the fluorescence of 2′,7′- dichlorofluorescein diacetate (DCFH-DA) as described previously [10]. In experiments using FCCP and Valinomycin (purchased from Sigma-Aldrich, St. Louis, MO, USA), cells were pre-incubated for the indicated time points with uncouplers. Cell samples were analyzed by using a fluorometer (Wallac 1420 Victor multilabel counter, Wallac Oy, Turku, Finland) with 485 nm excitation and 535 nm emission wavelenghts.

### Viability assay

Cell toxicity and apoptosis induction upon mitochondrial uncoupler treatment was measured using the CytoTox-Fluor™ Cytotoxicity Assay and Caspase 3/7 Assay (Promega, Madison, USA) as indicated in the manufactures protocol. In brief, one thousand human or mouse HSCs were plated in dark 96 well plates and treated with increasing concentrations of FCCP, Valinomycin or cycloheximide (Sigma-Aldrich). After 2 and 24 hours, cells were loaded with the cytotoxicity fluorescent marker bis-alanylalanyl-phenylalanyl-rhodamine 110 and incubated at 37°C for 30 minutes. Following, fluorescence was quantified (Wallac 1420). Subsequently, cells were lysed and loaded with a luminogenic caspase-3/7 substrate and incubated for more 30 minutes at room temperature, followed by quantification of light emission as a function of caspase 3/7 activity.

### Cell proliferation assay

Cell proliferation was analyzed by measuring DNA synthesis with the Click-it EdU Cell Proliferation Assay Kit (Invitrogen). 3,750 cells per cm^2^ were plated in the presence or absence of FCCP or Valinomycin in combination with Tin protoporphyrin IX dichloride (SnPP) (Tocris Bioscience, USA) from the first day of culture. After 24 hours, EdU labeling was initiated. After 18 or 48 hours, for human and mouse HSCs respectively, cells were formalin fixed and visualization of the EdU incorporation was obtained according to the manufacturer’s instructions.

### ATP levels measurement

ATP levels were determined using the ATP Determination Kit (Invitrogen). Briefly, 3000 cells/well were plated in white 96 well plates (Sigma, St. Louis, MO, USA). After two days in culture, cells were treated with FCCP and Valinomycin for 24 hours, followed by ATP determination as determined by manufacturer’s instructions.

### Statistical analysis

Statistical analyzes was performed using SPSS 16.0 (SPSS Inc., Chicago, USA). Data are expressed as mean ± standard mean. Differences among groups were analyzed for statistical significance by one-way ANOVA followed by Tukey. Results were considered significant when p < 0.05. All data shown are representative results of at least three independent experiments.

## Results

### Chemical uncoupling reduces ATP and ROS levels in HSCs while inducing genes involved in mitochondrial biogenesis

**Figure 1:**
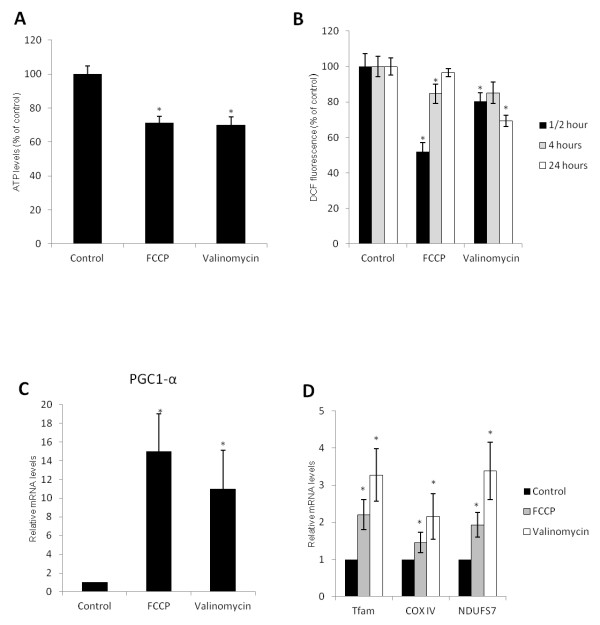
**FCCP and Valinomycin reduce HSC ATP levels, ROS production and stimulate mitochondrial biogenesis related genes.(A)** Cells were treated with 5 μM of FCCP or Valinomycin for 24 hours and ATP levels were measured using a luciferase based ATP determination kit. **(B)** ROS levels were analyzed in mHSCs treated for different time points with chemical uncouplers by DCF fluorescence. HSCs were treated with 5 μM of FCCP or Valinomycin for 7 days and the expression of genes involved in mitochondrial biogenesis PGC-1α **(C)**, Tfam, Ndufs7 and CoxIV **(D)** were analyzed by RT-qPCR. * indicates *P* < 0.05 compared to control groups. Data are expressed as means of 3 independent experiments ± SEM.

We determined whether FCCP and Valinomycin were able to reduce cellular ATP levels in freshly isolated mouse HSCs, one of the main features of chemical uncoupling. As shown in Figure 1A, 24 hours treatment with either FCCP or Valinomycin, two mitochondrial uncouplers with different modes of action, reduced 30% of ATP levels when compared to control cells. Next, we evaluated if uncouplers can modulate ROS, as they play a major role during HSC activation stimulating several pathways related to the activation process [13]. After 30 minutes, FCCP and Valinomycin reduced ROS levels to 50% and 80% of control cells respectively (Figure 1B). Interestingly, while FCCP shows a time dependent effect, Valinomycin shows no time dependent decrease in ROS levels, inducing a continuous reduction up to 24 hours. Following, we studied the effect of HSC uncoupling on genes related to mitochondrial biogenesis. These genes are stimulated when energy demand is high and have been shown to decrease ROS production and oxidative cell damage [14]. Peroxisomal proliferator activator receptor-γ coactivator-1α (Pgc-1α) is a regulator of mitochondrial biogenesis, interacting with several transcriptional factors, including mitochondrial transcription factor A (Tfam), a master regulator of mitochondrial biogenesis [15]. As shown on Figure 1C, treatment with FCCP and Valinomycin increased about 15 and 10 times the expression of Pgc-1α, respectively. Uncouplers also increased the expression of Tfam, NADH dehydrogenase [ubiquinone] iron-sulfur protein 7 (Ndufs7) and cytochrome c oxidase subunit IV (CoxIV), the last two being genes that encode proteins from the electron transport chain (Figure 1D). These results indicate that chemical uncoupling leads to mild reduction in ATP levels and reduction in ROS levels while stimulating transcription of mitochondrial biogenesis related genes in HSCs.

### Chemical uncouplers reduce the expression of pro-fibrogenic genes in HSCs

**Figure 2:**
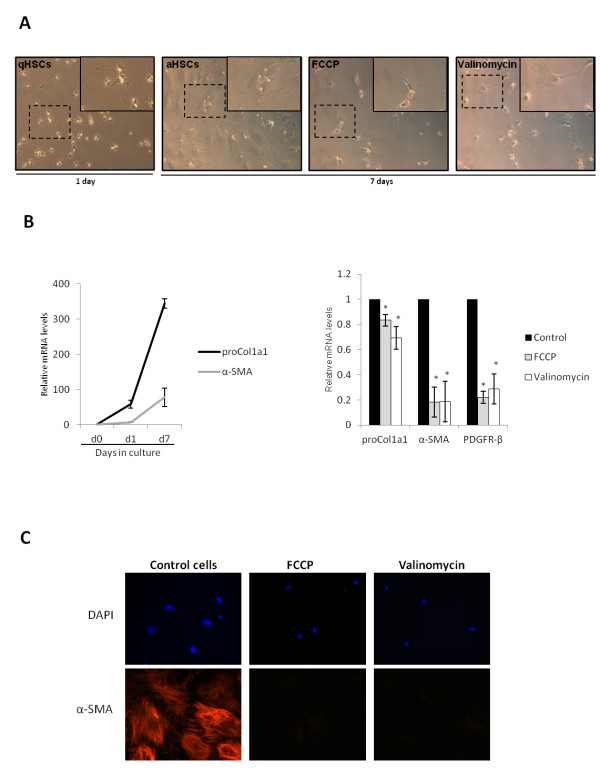
**FCCP and Valinomycin inhibit the expression of HSC pro-fibrogenic genes.(A)** Bright field images of mHSCs treated with FCCP (5 μM) or Valinomycin (5 μM) every two days for a period of seven days. qHSCs and aHSCs indicate quiescent and activated HSCs respectively. Dashed lines indicate the blow-up of area in inserts. **(B)** Expression of pro-fibrogenic genes during HSC activation *in vitro* and effect of uncoupler treatment, left and right graphics respectively. Mouse HSCs were treated for seven days with FCCP (5 μM) or Valinomycin (5 μM) and after this period mRNA levels of the indicated genes were analyzed by RT-qPCR. **(C)** α-SMA protein expression in cells treated with chemical uncouplers. Isolated mouse HSCs were cultivated on cover slips for 7 days and then fixed with 4% formaldehyde and stained with α-SMA antibodies. * indicates P < 0.05 compared to control groups. Data are expressed as means of 3 independent experiments ± SEM.

HSC activation is characterized by a phenotypic transformation from a small, lipid droplet containing cell, to a myofibroblastic phenotype, during which the cell loses its lipid droplets and acquires a large body size. The cell morphology of primary mouse HSCs treated for 7 days with chemical uncouplers was similar to that of freshly isolated quiescent HSCs (Figure 2A). As shown in the inserts, uncoupler treated cells presented a small round body size while control cells showed the typical activated myofibroblastic phenotype after 7 days of cell culture.

HSC activation is accompanied by the increased expression of pro-fibrogenic genes that are involved in several characteristics of the myofibroblastic HSC such as cell contraction and collagen deposition (Figure 2B, left graph). Uncoupler-treated cells showed a reduction of approximately 80% in the expression of α-SMA, a standard marker of HSC activation, and Pdgfrβ, a receptor involved in HSC proliferation (Figure 2B, right graphic). Next, we evaluated by immunohistochemistry if α-SMA protein expression was reduced in mouse HSCs treated with FCCP or Valinomycin. As seen in Figure 2C, α-SMA protein expression is down regulated by both uncoupler treatments.

### Mild chemical uncoupling does not induce cytotoxic effects or apoptosis

**Figure 3:**
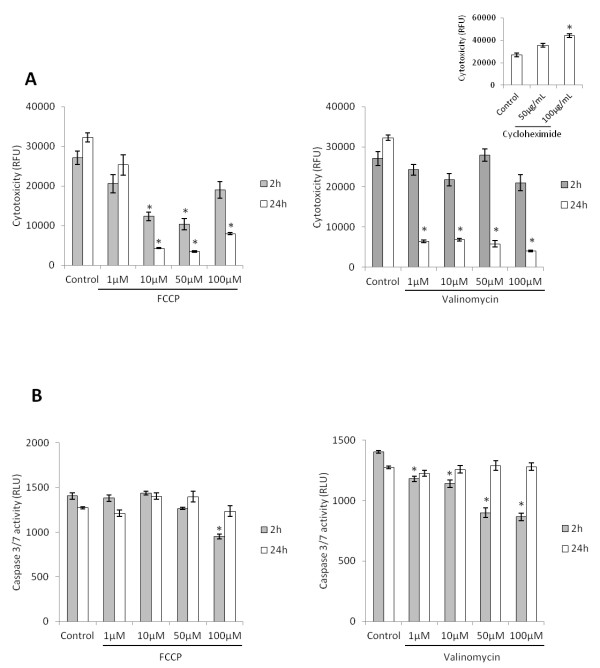
**Chemical uncoupling does not induce cytotoxicity on concentrations that inhibit HSC *in vitro* activation.(A)** Cytotoxic effect of mitochondrial uncouplers, as measured by fluorescence of bis-alanylalanyl-phenylalanyl-rhodamine 110, a peptide substrate of cytoplasmic peptidases. Insert shows cells treated with cycloheximide as positive control **(B)** Caspase 3/7 activity after treatment of cells with different concentrations of Valinomycin and FCCP on different time points. Enzyme activity was measured as described in material and methods. * indicates *P* < 0.05 compared to control group.

In order to test whether the concentrations of uncouplers used to inhibit HSC activation could be eliciting cytotoxicity, we treated cells with a wide concentration range of FCCP and Valinomycin and analyzed a possible induction of necrosis by addition of a fluorescent substrate of cytoplasmic endopetidases. As demonstrated in Figure 3A, FCCP and Valinomycin did not elicit necrosis on any concentration tested. Actually, FCCP and Valinomycin protected against cell death on most tested concentrations. Additionally, we observed no increase in apoptosis, as measured by caspase 3/7 activity, even with concentrations 10–20 fold higher than the one used to inhibit HSC activation (Figure 3B).

### FCCP inhibits HSC proliferation through activation of heme-oxygenase 1

**Figure 4:**
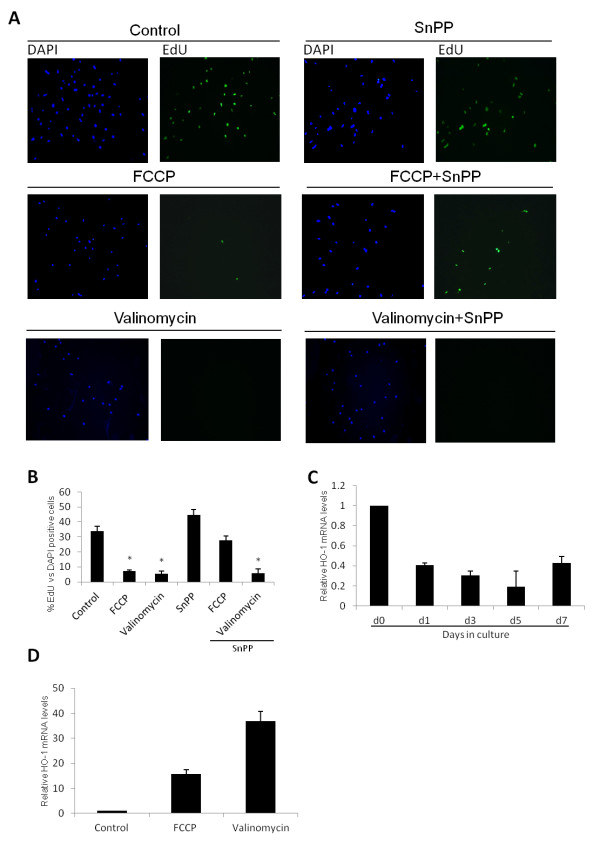
**FCCP inhibits HSC proliferation through HO-1.(A)** Representative images of cells stained with DAPI (blue fluorescence, left column) and EdU, as a measurement of DNA synthesis (green fluorescence, right column) at day 5. Cells were treated for 5 days with uncouplers (5 μM of each) alone or in combination with the heme-oxygenase inhibitor SnPP as indicated. **(B)** Quantification of proliferation (EdU positive) as percentage of DAPI positive cells visualized in **(A).** (**C**) HO-1 gene expression determined by RT-qPCR during *in vitro* activation of primary mouse HSCs and comparing 7 day old control cells versus cells treated for 7 days with FCCP or Valinomycin **(D)**. Data are expressed as means of 3 independent experiments ± SEM.

HSC proliferation is characteristic of HSC activation and a key feature in the progression of liver fibrosis. Hence, we investigated whether chemical uncoupling could reduce cell proliferation in HSCs. FCCP and Valinomycin treatment drastically decreased DNA incorporation of EdU, a BrdU analogue (Figure 4A). While control cells showed about 30% EdU positive cells during *in vitro* activation, FCCP and Valinomycin treated cells showed only 8% EdU positive cells, indicating less proliferation (Figure 4B).

Heme oxygenase 1 (HO-1) is the rate limiting enzyme in the degradation of heme groups and decreases cell proliferation when induced during HSC activation [16]. Therefore, we were interested in studying its expression during culture induced activation and its possible participation in the effect of both chemical uncouplers. HO-1 gene expression is down regulated during *in vitro* HSC activation and its expression can be rescued by FCCP and Valinomycin treatment (Figure 4C and D, respectively). To test if HO-1 plays a role in the anti-proliferative effect of the uncouplers we co-treated cells with Sn-protoporphyrin IX (SnPP, 5 μM), an inhibitor of HO-1. Co-treatment partially abrogated FCCP inhibition of proliferation (Figure 4A and 4B). Curiously, the observed Valinomycin effect was not affected by SnPP co-treatment, indicating that HO-1 does not play a role in the anti-proliferative effect of this chemical uncoupler.

### Induction of pro-fibrotic genes by TGF-β is inhibited by chemical uncoupling

**Figure 5:**
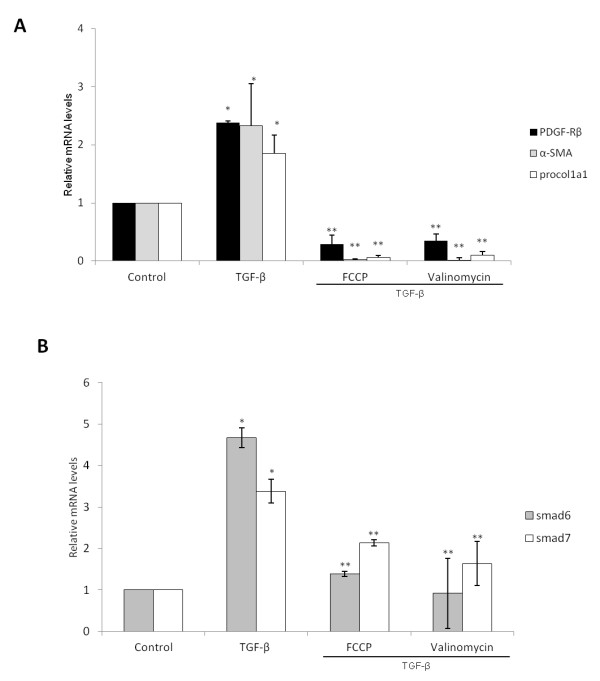
**FCCP and Valinomycin inhibit TGF-β signaling in 2 day old mHSCs.(A)** Expression of pro-fibrogenic genes in cells treated for 24 hours with 5 μM of FCCP and Valinomycin or together with TGF-β (10 ng/mL) as indicated. **(B)** Expression of TGF-β early immediate genes Smad6 and Smad7 on HSCs treated for 2 hours with TGF-β. * indicates *P* < 0.05 compared to control groups and ** indicates *P* < 0.05 compared to TGF-β group. Data are expressed as means of 3 independent experiments ± SEM.

TGF-β signalling induces several pro-fibrogenic genes in HSCs, participating in the activation process and liver fibrosis. Mitochondrial uncoupling is able to inhibit several signalling pathways in different cell types; hence we tested if mitochondrial uncoupling could affect TGF-β signalling in HSCs. A 24 hour treatment of 2 day old HSCs with TGF-β induced the expression of α-SMA, Procollagen 1a1 and Pdgfrβ, while in cells co-treated with Valinomycin and FCCP this TGF-β induced effect was largely inhibited (Figure 5A).

In order to analyze whether uncouplers directly affect TGF-β signalling, we studied if early immediate responsive genes for TGF-β, namely Smad6 and 7, were also being inhibited. As shown in Figure 5B, while TGF-β alone induced the expression of both genes after 2 hours of treatment, FCCP and Valinomycin inhibited such up-regulation, suggesting that both uncouplers affect TGF-β signalling.

### Mitochondrial uncouplers reduce activation of human HSCs

**Figure 6:**
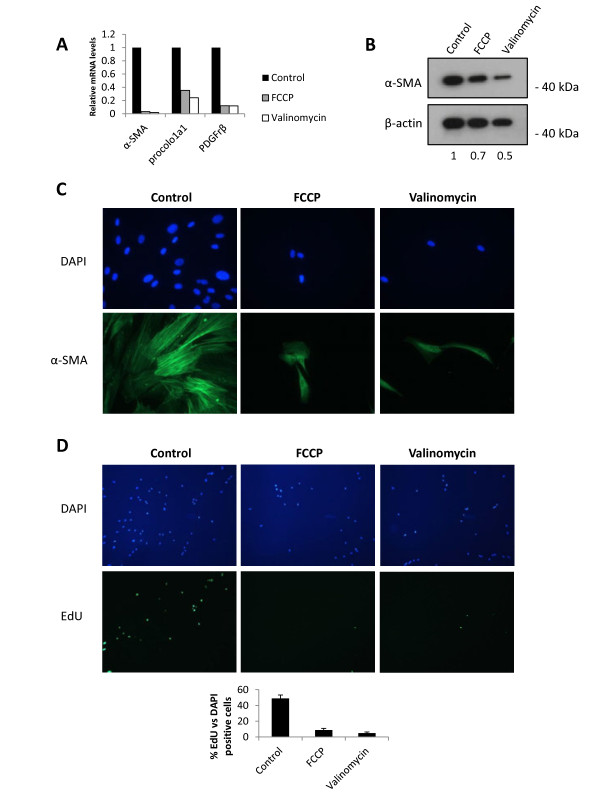
**Mitochondrial uncouplers reduce activation markers and proliferation of human derived HSCs.(A)** Human activated HSCs (passage 7) were treated for 5 days with FCCP or Valinomycin (10 μM, both compounds) followed by analysis of mRNA levels of pro-fibrogenic genes by RT-qPCR. Activated human HSCs treated for 5 days with mitochondrial uncouplers present reduced α-SMA protein expression as observed by western blot **(B)** and immunocytochemistry **(C)**. Numbers below Figure B represent densitometry values of α-SMA compared to control and normalized by β-Actin levels. **(D)** Representative images of cells stained with DAPI (blue fluorescence, left column) and EdU, as a measurement of DNA synthesis (green fluorescence, right column) at day 5. **(B)** Quantification of proliferation (EdU positive) as percentage of DAPI positive cells visualized in **(D)**.

We next evaluated if mitochondrial uncoupling was also able to reduce activation of primary human HSCs. First, we tested if human derived HSCs presented any cytotoxicity upon uncoupler treatment. High concentrations, tenfold higher than the one used to inhibit HSC activation, did not elicit increased cytotoxicity as measured by leakage of cytoplasmic peptidases and induced activation of caspase 3/7 only after 24 hours treatment (Additional file 1: Figure S1). Secondly, as shown on Figure 6A, both uncouplers induce a robust decrease in the expression of pro-fibrogenic genes after 5 days of treatment. A marked inhibition of α-SMA protein expression was also observed for both treatments (Figure 6B, C). As with primary mouse HSCs, we observed a decrease in the incorporation of EdU in FCCP and Valinomycin treated human HSCs (Figure 6D), indicating a decrease in cell proliferation. These results suggest that mitochondrial uncoupling cannot only reduce culture induced activation of mouse HSCs, but also reduces the fibrogenic phenotype of human activated HSCs.

## Discussion

Patients with NAFLD present mitochondrial ultrastructural alterations that might reflect mitochondrial impairment and subsequent hepatocyte apoptosis [17]. Although there is mounting evidence for a mitochondrial role during liver disease, few studies have focused on the role of this organelle during HSC activation [18,19]. To our knowledge, this is the first demonstration that mild mitochondrial uncoupling can reduce several aspects of HSC activation.

Mitochondria can elicit cell signalling and influence cell function by adapting metabolism, altering cell response to cytokines and nuclear gene expression [20]. Although initially counter intuitive, several studies indicate that uncoupling of the mitochondrial electron transport chain can elicit beneficial cell adaptations and improve several pathologies. Heart ischemia/reperfusion detrimental effects can be ameliorated through pre-conditioning with mild uncoupling using chemical uncouplers while studies on mice show that uncoupler treatment can increase life span, reduce oxidative stress and improve insulin levels [21]. Remarkably, a study by Rohas *et al*. has shown that once mild uncoupling is set, cells trigger a compensatory mechanism where PGC-1α is activated and orchestrate a signalling cascade to compensate the reduction in ATP levels [22]. We also observe an increase in PGC-1α expression during HSC mild uncoupling and a protective effect against cell necrosis and apoptosis in both human and mouse derived HSCs.

ROS and consequent oxidative stress participate in HSC activation through several mechanisms such as glutathione depletion and activation of transcription factors [23]. Indeed, the collagen type I promoter can be regulated by ROS generation in HSCs, demonstrating a fundamental role for oxidative stress in liver fibrosis perpetuation [24]. We observed a decrease in ROS levels in cells treated with uncouplers, suggesting ROS reduction as one possible mechanism by which these molecules inhibit HSC activation. Additionally, this result also indicates that treatment with FCCP and Valinomycin induces only mild uncoupling, since harmful uncoupling is associated with high amounts of ROS and oxidative stress [25,26]. Curiously, FCCP and Valinomycin show different antioxidant efficiencies. Valinomycin had a stable and less efficient effect on reducing ROS levels, while FCCP reduced ROS drastically after 30 minutes, reaching control levels after 24 hours. FCCP and Valinomycin uncouple mitochondria by different mechanisms. While FCCP is a proton mobile carrier that acidifies the mitochondrial matrix, Valinomycin acts as a specific potassium ionophore leading to alkalinisation of the mitochondrial matrix. This different effect on matrix pH can influence the dissimilar modulation of ROS levels [27]. Since both chemical uncouplers affect ATP levels by the same extend, it also indicates that the mode by which they affect matrix pH does not influence the net result on mitochondrial uncoupling.

Transcriptional regulation by the so-called adipogenic transcription factors is essential to keep HSC quiescence and has been shown to, once stimulated, inhibit several aspects of HSC activation [28]. Hence, it is hypothesized that signalling pathways leading to adipocyte differentiation similarly act on HSC activation [29]. Brown adipocytes also share analogous signalling pathways during differentiation, but opposite to white adipocytes, they present small disperse lipid droplets in the cytoplasm and activate mitochondrial uncoupling when thermogenesis is needed. We show that chemical uncoupling inhibits HSC activation and consequently stimulates the maintenance of the quiescent phenotype. Curiously, treatment of white adipocytes with chemical uncouplers induces the opposite of what is seen in HSCs, stimulating de-differentiation, (*i.e.* loss of lipid droplet and inducing a fibroblastic phenotype) [8]. Moreover, AMP-activating kinase (AMPK), a kinase with a main role in maintaining cellular energy levels, induces differentiation of brown adipocytes when chronically activated, while it inhibits the differentiation of white adipocytes [30]. In HSCs, AMPK inhibits several aspects of HSC activation and stimulates quiescent characteristics [31]. Together, these data and the results observed in this study indicate that the HSC activation process shares analogous signalling pathways with the differentiation of brown adipocytes, cells that present mitochondrial uncoupling activity and, more importantly, a lipogenic phenotype similar to the one of quiescent HSCs.

We show that different aspects of human and mouse HSC activation such as α-SMA expression and proliferation are inhibited by chemical mitochondrial uncoupling. One important aspect of activated HSC is the capacity to proliferate and consequently aggravate chronic liver injury. HO-1, an enzyme involved in the metabolism of heme, can reduce HSC proliferation mainly due to the production of bilirubin, an antioxidant end product of heme group degradation [16]. Curiously, both uncouplers inhibit the down regulation of HO-1 seen during activation, but HO-1 is only responsible for the anti-proliferative effect of FCCP and not Valinomycin, as shown using the HO-1 inhibitor SnPP. It has been reported that Valinomycin is able to inhibit the proliferation of different cell lines, although the mechanism is still not fully understood [32]. Our results show that, in general, uncoupling can inhibit HSC proliferation, but the mechanism diverges depending on the mode of mitochondrial uncoupling.

TGF-β plays an essential role during liver fibrosis, and has been intensely investigated as a target for therapy. This cytokine activates HSC *in vivo*, and TGF-β KO models have shown a dramatic reduction in α-SMA positive cells in liver fibrosis with a consecutive decrease in collagen deposition [33]. In this study we observed an increase in the expression of several fibrogenic genes when HSCs were treated with TGF-β which was completely abrogated by co-treatment with chemical uncouplers. The inhibition of TGF-β induced early-immediate genes Smad6/7 by the uncouplers suggests a direct influence on TGF-β signalling. Although it is not clear how mild mitochondrial uncoupling can influence TGF-β signalling, it is known that this cytokine can induce the release of mitochondrial calcium stores and that this process is necessary for the activation of protein kinases and downstream signalling of TGF-β [34]. Chemical uncoupling can influence calcium release from mitochondria [35] and therefore might influence TGF-β signalling through modulation of mitochondrial calcium stores.

Intriguingly, the observations in this study add a new perspective to the action of some molecules that are already known to inhibit HSC activation. For example, curcumin has been shown to be a powerful mitochondrial uncoupler in the same concentration range known to inhibit HSC fibrogenic features [36,37].

## Conclusion

The present study shows, for the first time, that mild mitochondrial uncoupling can inhibit HSC activation. Importantly, we also observed a reduction in fibrogenic features of human derived HSCs. Contrarily to mouse HSCs, which were treated with FCCP and Valinomycin still at the quiescent phenotype, the human HSCs used in this study presented an activated phenotype when treated (passage 7). Nonetheless, we still observed a decrease in fibrogenic features on both gene and protein levels. These last results show that mild mitochondrial uncoupling, additionally to its capacity to inhibit the first steps of HSC activation, is able to induce a decrease in activation features of already myofibroblastic HSCs. This is an important characteristic when considering possible applications in the treatment of liver fibrosis. In summary, we show that mild mitochondrial uncoupling can inhibit several aspects of HSC activation and indicates HSC mitochondrial metabolism as a possible new target for liver fibrosis therapy.

## Competing interests

The authors declare that there is no duality of interest associated with this manuscript.

## Authors’ contributions

ELG: study concept, experimental design, acquisition of data, drafting of the manuscript. JB and LD: acquisition and isolation of human HSCs. MN and ES: acquisition of human liver donors and liver material; LAvG: study supervision, experimental design and critical revision of the manuscript. All authors read and approved the final manuscript.

## Pre-publication history

The pre-publication history for this paper can be accessed here:

http://www.biomedcentral.com/1471-230X/12/68/prepub

## Supplementary Material

Additional file 1 **Figure S1.** Mitochondrial uncouplers show no cytotoxicity at low concentrations. (A) Cytotoxic effect of mitochondrial uncouplers on human HSCs, as measured by fluorescence of bis-alanylalanyl-phenylalanyl-rhodamine 110, a peptide substrate of cytoplasmic peptidases. (B) Caspase 3/7 activity after treatment of cells with different concentrations of Valinomycin and FCCP on different time points. Enzyme activity was measured as described in material and methods. * indicates *P* < 0.05 compared to control group. Click here for file
