# Supplementary figures and images for: Mitochondrial uncouplers inhibit hepatic stellate cell activation
Source: BMC Gastroenterol. 2012 Jun 11;12:68. doi: 10.1186/1471-230X-12-68 (PMC3439697; doi:10.1186/1471-230X-12-68)

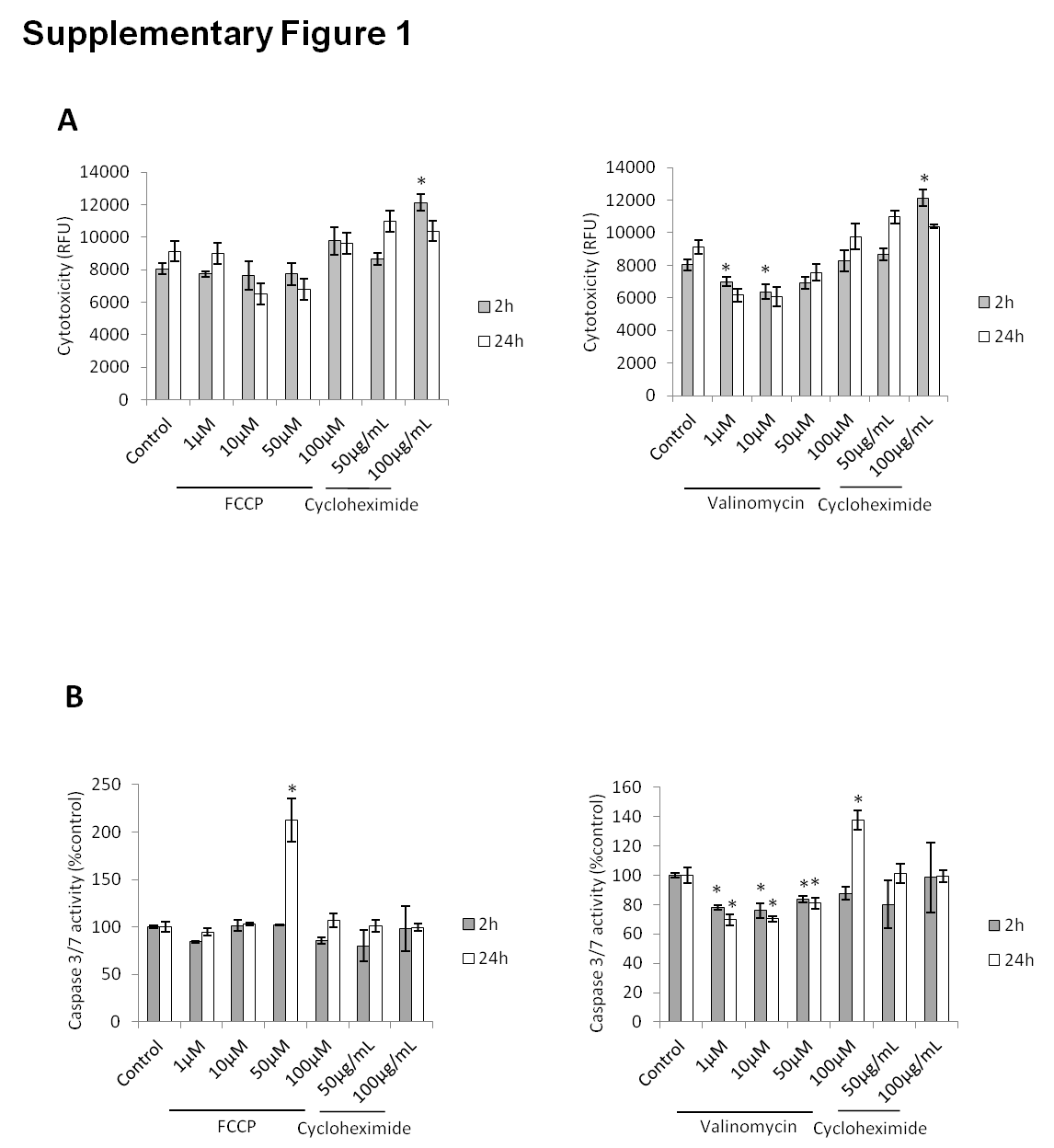

Supplement: Additional file 1 — Figure S1. Mitochondrial uncouplers show no cytotoxicity at low concentrations. (A) Cytotoxic effect of mitochondrial uncouplers on human HSCs, as measured by fluorescence of bis-alanylalanyl-phenylalanyl-rhodamine 110, a peptide substrate of cytoplasmic peptidases. (B) Caspase 3/7 activity after treatment of cells with different concentrations of Valinomycin and FCCP on different time points. Enzyme activity was measured as described in material and methods. * indicates P < 0.05 compared to control group. [file 1471-230X-12-68-S1.tiff]
